# Ritanserin suppresses acute myeloid leukemia by inhibiting DGKα to downregulate phospholipase D and the Jak-Stat/MAPK pathway

**DOI:** 10.1007/s12672-023-00737-9

**Published:** 2023-07-01

**Authors:** Jinshui Tan, Mengya Zhong, Yanyan Hu, Guangchao Pan, Jingwei Yao, Yuanfang Tang, Hongpeng Duan, Yuelong Jiang, Weihang Shan, Jiaqi Lin, Yating Liu, Jiewen Huang, Huijian Zheng, Yong Zhou, Guo Fu, Zhifeng Li, Bing Xu, Jie Zha

**Affiliations:** 1 Department of Hematology, The First Affiliated Hospital of Xiamen University and Institute of Hematology, School of Medicine, Xiamen University, Xiamen, 361003 Fujian People’s Republic of China; 2 Key Laboratory of Xiamen for Diagnosis and Treatment of Hematological Malignancy, No. 55, Shizhen Hai Road, Xiamen, 361003 Fujian People’s Republic of China; 3 State Key Laboratory of Cellular Stress Biology, School of Life Sciences, Innovation Center for Cell Biology, Xiamen University, Xiamen, 361002 Fujian China; 4 School of Pharmaceutical Sciences, Xiamen University, Xiamen, 361002 Fujian China; 5 The School of Clinical Medicine, Fujian Medical University, Fuzhou, 350122 Fujian China; 6 State Key Laboratory of Cellular Stress Biology, School of Medicine, Innovation Center for Cell Biology, Xiamen University, Xiamen, 361002 Fujian China

**Keywords:** Ritanserin, R/R AML, DGKα, Jak-Stat, MAPK

## Abstract

Refractory or relapsed (R/R) AML is the most challenging form of AML to treat. Due to frequent genetic mutations, therapy alternatives are limited. Here, we identified the role of ritanserin and its target DGKα in AML. Several AML cell lines and primary patient cells were treated with ritanserin and subjected to cell proliferation, apoptosis and gene analyses with CCK-8 assay, Annexin V/PI assay and Western blotting, respectively. We also evaluated the function of the ritanserin target diacylglycerol kinase alpha (DGKα) in AML by bioinformatics. In vitro experiments have revealed that ritanserin inhibits AML progression in a dose- and time-dependent manner, and it shows an anti-AML effect in xenograft mouse models. We further demonstrated that the expression of DGKα was elevated in AML and correlated with poor survival. Mechanistically, ritanserin negatively regulates SphK1 expression through PLD signaling, also inhibiting the Jak-Stat and MAPK signaling pathways via DGKα. These findings suggest that DGKα may be an available therapeutic target and provide effective preclinical evidence of ritanserin as a promising treatment for AML.

**Supplementary Information:**

The online version contains supplementary material available at 10.1007/s12672-023-00737-9.

## Introduction

The frequency of acute myeloid leukemia (AML) has been increasing as the population ages, and it is a biologically complex and molecularly heterogeneous disease. Clinically, refractory or relapsed (R/R) AML is the most challenging form of AML to treat, with a very dismal prognosis [1]. It is reported that the five-year overall survival (OS) of patients with R/R AML is 10%, and the median overall survival (OS) is about 6 months [2]. Even in patients who meet the strict chemotherapy conditions, the complete remission (CR) rate and OS are not satisfactory [3]. Although there have been major advances in our knowledge of molecular pathogenesis, clinical trials are still the best way to determine the standard treatment for R/R AML [1, 4]. Therefore, there is an urgent need to find novel therapies to improve the treatment efficiency of R/R AML.

Initially developed as a serotonin receptor antagonist, ritanserin has undergone clinical trials for applications including schizophrenia, alcoholism, and insomnia [5–7]. Recent studies have noted its function as a particular inhibitor of diacylglycerol (DAG) kinase α (DGKα), which can inhibit the progression of certain cancers and enhance immunotherapies [8]. Ritanserin is cytotoxic against various tumors through putative downstream targets of DGKα, including mammalian target of rapamycin [9], hypoxia-inducible factor 1-α [9], geranylgeranyl transferase I [10] and other kinases involved in mitogen-activated protein kinase (MAPK) signaling [11, 12]. Although ritanserin was never put forward for FDA approval, its high oral bioavailability and lack of serious side effects have caused it to gain widespread attention, and many studies on ritanserin are being conducted [11, 13]. Considering that AML is a complex and incurable disease with adverse clinical outcomes, it may be a feasible choice to reuse ritanserin as a DGKα inhibitor for tumor indications.

Here, we identified the role of ritanserin and its target DGKα in AML. In vitro experiments have revealed that ritanserin inhibits AML progression by inhibiting cell proliferation and inducing apoptosis, and an anti-AML effect has been observed in xenograft mouse models. To pinpoint the specific function and molecular mechanisms of DGKα in AML, we analyzed the underlying biological mechanisms and their potential as prognostic factors by bioinformatics. Interestingly, this study showed that ritanserin, a DGKα-targeted inhibitor, may participate in the phospholipase D (PLD) signaling pathway, also negatively regulating the Jak-Stat and MAPK signaling pathways and exerting anti-AML tumor activity.

## Materials and methods

### Cell lines and reagents

The human AML cell lines Kasumi-1 and KG-1α were provided by the Department of Hematology, The First Affiliated Hospital of Xiamen University (Fujian, China) and both cell lines were tested and identified. 10% fetal bovine serum (FBS, Excell Bio, Shanghai, China), 100 units/ml penicillin and 100 μg/ml streptomycin (Invitrogen, MA, USA) were added to RPMI-1640 medium (Basal Media, Shanghai, China) for cell culture. Both cell lines were maintained at 37° C in a 5% CO_2_ incubator. Ritanserin (T16759) was provided by TargetMol (TargetMol, MA, USA) and dissolved in dimethyl sulfoxide (DMSO, Sigma, MO, USA). When administered in mice, the compounds were diluted with 0.3%(w/v) CMC-Na suspension and injected intraperitoneally.

### Cell viability assay

AML cells (1 × 10^4^ per well) were seeded in 100 μl medium in 96-well plates. The cell viability was determined by cell counting kit -8(CCK-8, TargetMol) after 24, 48 and 72 h of treatment with DMSO or ritanserin with specific concentration. Three replicates were presented and the results were expressed as the percentage of living cells compared with the control group. GraphPad Prism 8 was used for statistical analysis and image rendering.

### Analysis of apoptosis

As described above, cells were cultured and exposed to Ritanserin at the specified concentration for 24, 48 and 72 h. The annexin V/PI apoptosis detection kit (BD Pharmingen, USA) and NovoCyte Quanteon flow cytometry (ACEA Biosciences, CA, USA) were used to analyze cells. Annexin V positive cells were defined as apoptotic cells, and the results of three repetitions were presented as the mean ± SD.

### Quantitative real-time PCR (qRT-PCR)

According to the manufacturer's instructions, the total RNA was extracted by SteadyPure universal RNA extraction kit (AG21017, Accurate Biology, Hunan, China), and then reverse transcribed into cDNA using Evo M-MLV RT Master Mix (AG11706, Accurate Biology). qRT‒PCR was performed with a SYBR Green Premix Pro Taq HS qPCR Kit (AG11702, Accurate Biology) and then amplified and detected by a Light Cycler 480 System (Roche, Basel, Switzerland). The primer sequences used were as follows: DGKA forward 5’-CACCCACCCACTTCTCGTCTTTG-3’, reverse 5’- CGGAGCCCTATCTCAGGACCATC-3’; β-actin forward 5’-TGTGGCATCCACGAAACTAC-3’, reverse 5’-GGAGCAATGATCTTGATCTTCA-3’. Three replicate experiments were conducted, and the results showed the relative gene expression of β -actin.

### Western blot

The western blot experiment was carried out according to the description [14]. After separation by SDS-PAGE, the designated proteins were transferred to the PVDF membranes, and the PVDF membranes were horizontally cut based on the location of the target molecule. The following antibodies were used in this research: anti-DGKA (CA60796, 1:1000, Cell Signaling Technology, MA, USA), anti-Jak1 (CA29261, CST), anti-Jak3 (CA8863, CST), anti-Stat5 (CA25656, CST), anti-P-Stat5 (Tyr694) (CA72712, CST), anti-Stat3 (CA12640, CST), anti-P-Stat3 (Tyr705) (CA9145, CST), anti-PARP (CA9532, CST), anti-cleaved PARP (CA5625, CST), anti-caspase-3 (CA9662, CST), anti-MEK (CA8727, CST), anti-P-MEK (CA3958, CST), anti-ERK (CA4695, CST), anti-P-ERK (CA8544, CST), anti-GAPDH (CA5174, CST), anti-SphK1 (SP5421, 1:1000, ECM Biosciences, KY, USA), anti-P-SphK1 (Ser-225) (SP1641, ECM Biosciences) and HRP-linked anti-rabbit IgG (CA7074, CST). Finally, all protein were visualized by ECL Western blotting Detection Kit (GeneFlow, Staffordshire, UK). The protein agonists applied were as follows: SphK1 agonist K6PC-5(HY-124042, MedChemExpress); MEK/ERK agonists C16-PAF (HY-108635, MedChemExpress); JAK/STAT agonists RO8191 (T22142, TargetMol).

### Patients and primary AML samples

Twenty human primary AML and five normal hematopoietic stem cell specimens were collected from the First Affiliated Hospital of Xiamen University, Department of Hematology (Fujian, Xiamen). According to the Helsinki Declaration, this study was approved by the Ethics Review Committee of the First Affiliated Hospital of Xiamen University. Informed consent was obtained from all individual participants in this research. Ficoll-Hypaque density gradient column (Cytova, Uppsala, Sweden) was used to isolate monocytes. CD34^+^ primary AML cells (581, Biolegend, CA, USA) were sorted by a NovoCyte Quanteon Flow Cytometer (ACEA Biosciences).

### Bioinformatics analyses

TCGA (https://www.cancer.gov/), GTEx (https://www.gtexportal.org/) and GSE12417 data [15] were retrieved from published literature. TCGA, GTEx data and GSE12417 were analyzed using R studio software (version 1.2.1335). R software packages "survminer" and "survival" were used to calculate the cutoff value of survival curve and plot survival curve, respectively. Volcano and heatmap are drawn by using R software packages "ggplot" and "heatmap". The “performance analytics” and “corrplot” R packages were used to draw the correlation plot. GO, KEGG and GSEA pathway enrichment analyses were performed by the R package “clusterProfiler” [16, 17] and GSEA software version 4.2.3.

### AML xenograft in mice

All animal experiments were approved by the Ethics Committee of Xiamen University. After 1 Gy irradiation, 2 × 10^6^ Kasumi-1 cells were injected intravenously into NOD-PRKDC/IL-2RG/mice (6-week-old, female). A week later, the mice were randomized divided into two groups (8 animals per group) and received vehicle (0.3% sodium carboxymethyl cellulose) or ritanserin (5 mg/kg/day) administered by intraperitoneal injection for two successive weeks. The leukemia burden was determined by intravital imaging every week. After 14 days of treatment, 3 mice were sacrificed from each group. The spleen (SP) and bone marrow (BM) were extracted for flow cytometry, HE staining, and immunohistochemistry analysis. The leukemia infiltration level was detected according to the surface markers CD45 (HI30, Biolegend, CA, USA), CD34 (581, Biolegend), and mCD45 (563890, BD Biosciences, NJ, USA) by a NovoCyte Quanteon Flow Cytometer (ACEA Biosciences). For immunohistochemistry analysis, the tissue slices were incubated overnight with primary antibodies targeting CD34 (ab110643, 1:250, Abcam), CD45 (CA13917, 1:400, CST) and DGKA (CA60796, 1:400, CST) at 4 °C. Then, DAB (DAB-2032, MXB Biotechnologies, Fujian, China) was applied for 30 s for the chromogenic reaction. Analysis was performed under an automatic digital slide scanner, Zeiss AxioScan7 (Zeiss, BW, Germany).

### Statistical analyses

GraphPad Prism 8.0 was applied for statistical analysis. The mean ± SD was used to describe continuous variables. Two independent-sample t-test was used to analyze the differences between groups. Multiple group comparisons were made using one-way ANOVA. Results with the p-value less than 0.05 had statistical significance.

## Results

### Ritanserin impairs cell proliferation and induces apoptosis in AML

**Fig. 1:**
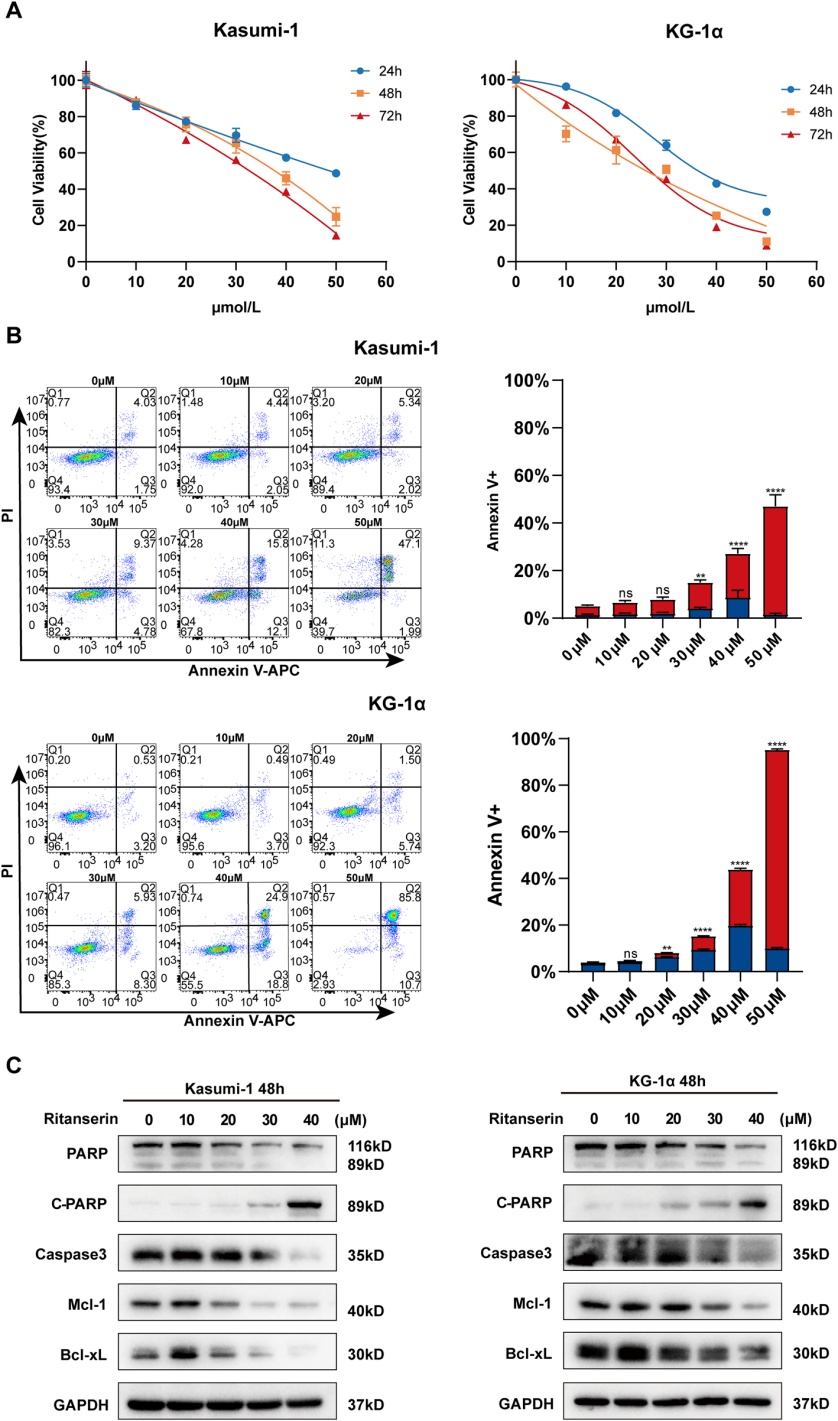
Ritanserin displays cytotoxic effects in AML cells. **A** The cell viability of Kasumi-1 and KG-1α cells were detected by CCK-8 assay after treating with increasing concentrations of ritanserin for 24, 48 and 72 h. **B** Kasumi-1 and KG-1α cells were exposed to Annexin V/PI double staining to detect the apoptosis ratio by ritanserin for 48 h. Data are presented as the mean ± S.D. of triplicate experiments. (**p < 0.01; ****p < 0.0001). **C** Western blotting was examined after exposure to ritanserin for 48 h in Kasumi-1 and KG-1α cells

First, we assessed the cytotoxicity of ritanserin against two human AML cell lines, Kasumi-1 and KG-1α. Ritanserin was applied to cells at a variety of concentrations for the specified periods, and CCK-8 assay results were obtained. As shown in Fig. 1A, ritanserin dramatically reduced the proliferative activity in AML cells in both a dose- and time-dependent manner. On the basis of cell viability, the IC_50_ values for each cell line at 24, 48, and 72 h were also computed and displayed concurrently (Table 1). In Kasumi-1 and KG-1α cells, the IC_50_ decreased with longer treatment times, as predicted, with values at 72 h (29.75 ± 0.47 µM and 25.88 ± 0.11 µM, respectively) being significantly less than those at 24 h (51.01 ± 0.62 µM and 37.7 ± 0.55 µM, respectively).

**Table 1:** IC_50_ of the AML cell treated with Ritanserin

Cell lines	IC_50_(μM)		
	24 h	48 h	72 h
Kasumi-1	50.01 ± 0.62	35.34 ± 2.17	29.75 ± 0.47
KG-1α	37.7 ± 0.55	23.57 ± 2.00	25.88 ± 0.11

The apoptosis of cells was detected to further evaluate the cytotoxic effect of ritanserin on AML. The degree of cell apoptosis was steadily increased in conjunction with the dose and duration of ritanserin treatment (Fig. 1B and Fig. S1). Combining the IC_50_ values of the cell proliferation assays, KG-1α cells were found to be more susceptible to ritanserin than Kasumi-1 cells. Given the crucial role that caspases play in the apoptosis execution mechanism, we wondered whether the activation of caspases was necessary for ritanserin-induced cell death. We examined the protein expression of caspase-3, PARP, and anti-apoptosis markers (Mcl-1 and Bcl-xL) through western blotting. Cleaved PARP was substantially increased after receiving ritanserin therapy after 48 h, indicating an apoptotic execution state. Moreover, there was a noticeable downregulation in the expression of total caspase-3, MCL1, and Bcl-xL (Fig. 1C). These results demonstrated that ritanserin effectively inhibited cell proliferation, and causes AML cells to undergo caspase-dependent apoptosis.

### Highly expressed DGKα reveals a poor prognosis for AML

**Fig. 2:**
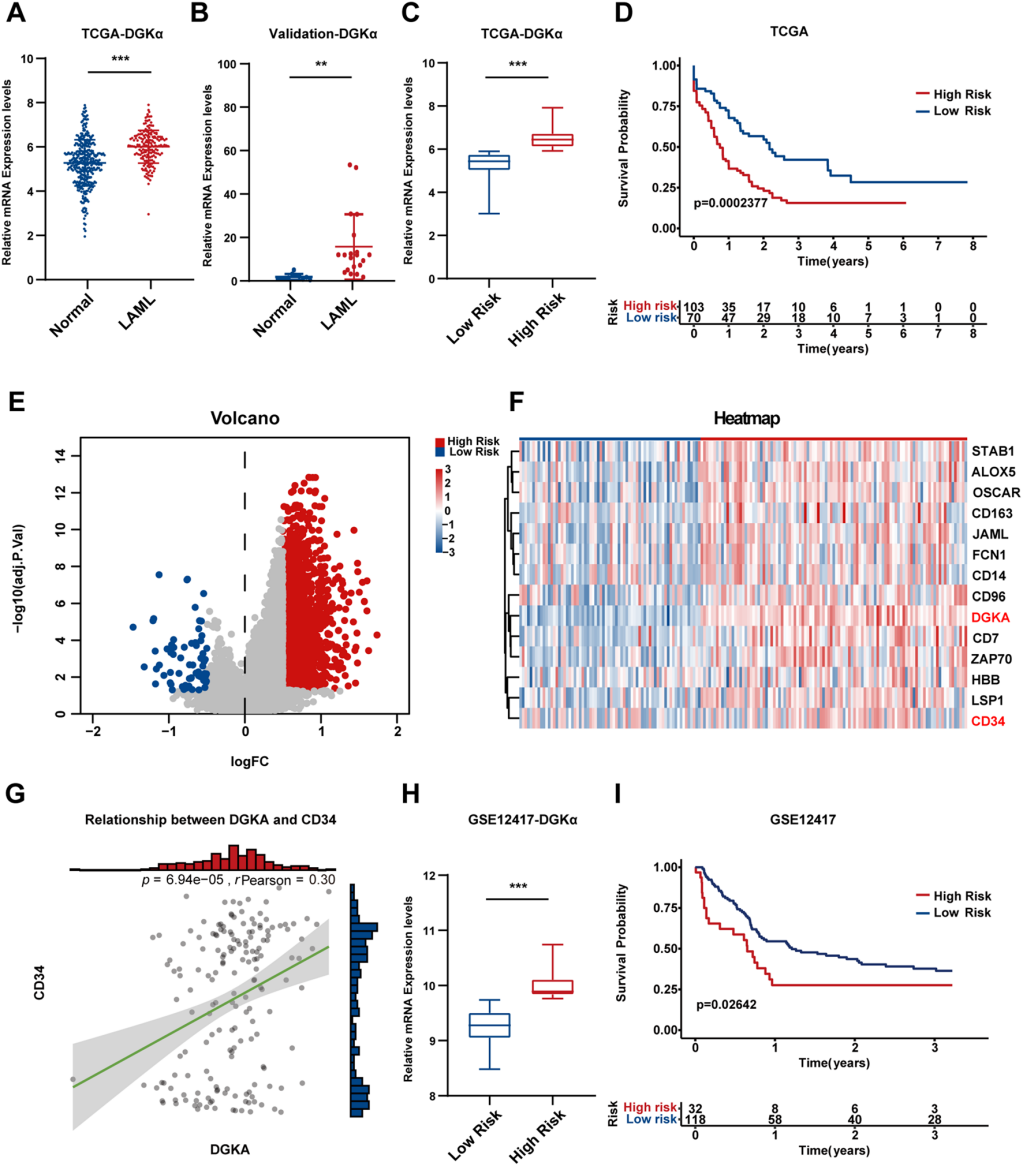
High DGKα expression in AML samples was associated with a worse prognosis. **A** The mRNA expression levels of DGKα from TCGA (n = 173) and GTEx datasets (n = 337). **B** DGKα mRNA levels in 20 AML samples and 5 normal peripheral blood samples were measured by RT‒qPCR analysis. Risk stratification of DGKα expression in **C** TCGA AML samples and **H** GSE12417. The overall survival of the high- and low-risk groups in **D** TCGA and **I** GSE12417. **E** The volcano plot shows 2273 and 70 genes upregulated and downregulated significantly in the high-risk group compared to the low-risk group, respectively. **F** Heatmap of different genes in the high-risk and low-risk groups. **G** Correlation between DGKα and CD34 in AML samples in TCGA. (**p < 0.01; ***p < 0.001)

Since ritanserin has recently been shown to exert powerful antitumor effects as a DGKα specific inhibitor, we sought to explore the role of DGKα in AML. In the TCGA and GTEx datasets, we examined the expression of DGKα in normal peripheral blood samples (n = 337) and AML samples (n = 173). DGKα expression was markedly elevated in AML samples compared to normal samples (Fig. 2A). We obtained blood samples of bone marrow from AML patients and analyzed the mRNA expression level of DGKα, and the results were found to be compatible with the public datasets (Fig. 2B), thus confirming the upregulation of DGKα in AML.

We further divided 173 AML samples into high-risk and low-risk groups according to the expression level of DGKα and discovered that the high-risk group had worse outcomes (Fig. 2C, D). Additionally, we discovered the differentially expressed genes (DEGs) in the two groups. In total, 2273 and 70 genes were substantially upregulated and downregulated in the high-risk group, respectively (Fig. 2E, logFC ≥ 0.5, P < 0.05). Interestingly, we discovered that the high-risk group had higher levels of CD34 expression (Fig. 2F). In the AML samples, correlation analysis revealed that DGKα expression was positively correlated with CD34 (Fig. 2G). To verify the significance of DGKα in TCGA, we also investigated the survival function of DGKα in GSE12417 (n = 150). The high-risk group was associated with unfavorable survival (Fig. 2H, I). Collectively, these data indicated that high expression of DGKα in AML was related to the poor prognosis, which also suggested that the anti-leukemia effect of ritanserin is partly due to the inhibition of DGKα.

### Biological function and pathway analysis in AML

As a pro-oncogene, DGKα promotes proliferation and anti-apoptosis in leukemia [18, 19]. To elucidate the molecular mechanism of ritanserin in AML, we continued to explore the potential biological function of DGKα. Through Gene Ontology (GO) analysis of AML samples, the DEGs in the high-risk group (high DGKα expression) in AML were found to be primarily involved in phospholipid binding, DAG kinase activity, and NAD^+^ kinase activity and to promote phospholipid, DAG, and glycerophospholipid metabolic processes (Fig. S2A). According to the Kyoto Encyclopedia of Genes and Genomes (KEGG) enrichment analysis, AML, phospholipase D (PLD), MAPK and Jak-Stat signaling pathways were the major biological processes affected by DEGs in the two groups (Fig. S2B). Additionally, Gene Set Enrichment Analysis (GSEA) enrichment demonstrated that the high-risk group was implicated in the development of leukemia and the metabolic pathways connected to PLD signaling (Fig. S2C–F). Consistent with the KEGG results, two crucial pathways, the MAPK and Jak-Stat signaling pathways, were enriched in the high-risk group (Fig. S2G, H). Altogether, our findings showed that in the high-risk group (high DGKα expression), DGKα regulated the PLD and Jak-Stat/MAPK signaling pathways, which are highly linked with a worse prognosis, to impact the carcinogenesis of AML.

### Ritanserin affects PLD signaling and regulates SphK1 expression via DGKα

**Fig. 3:**
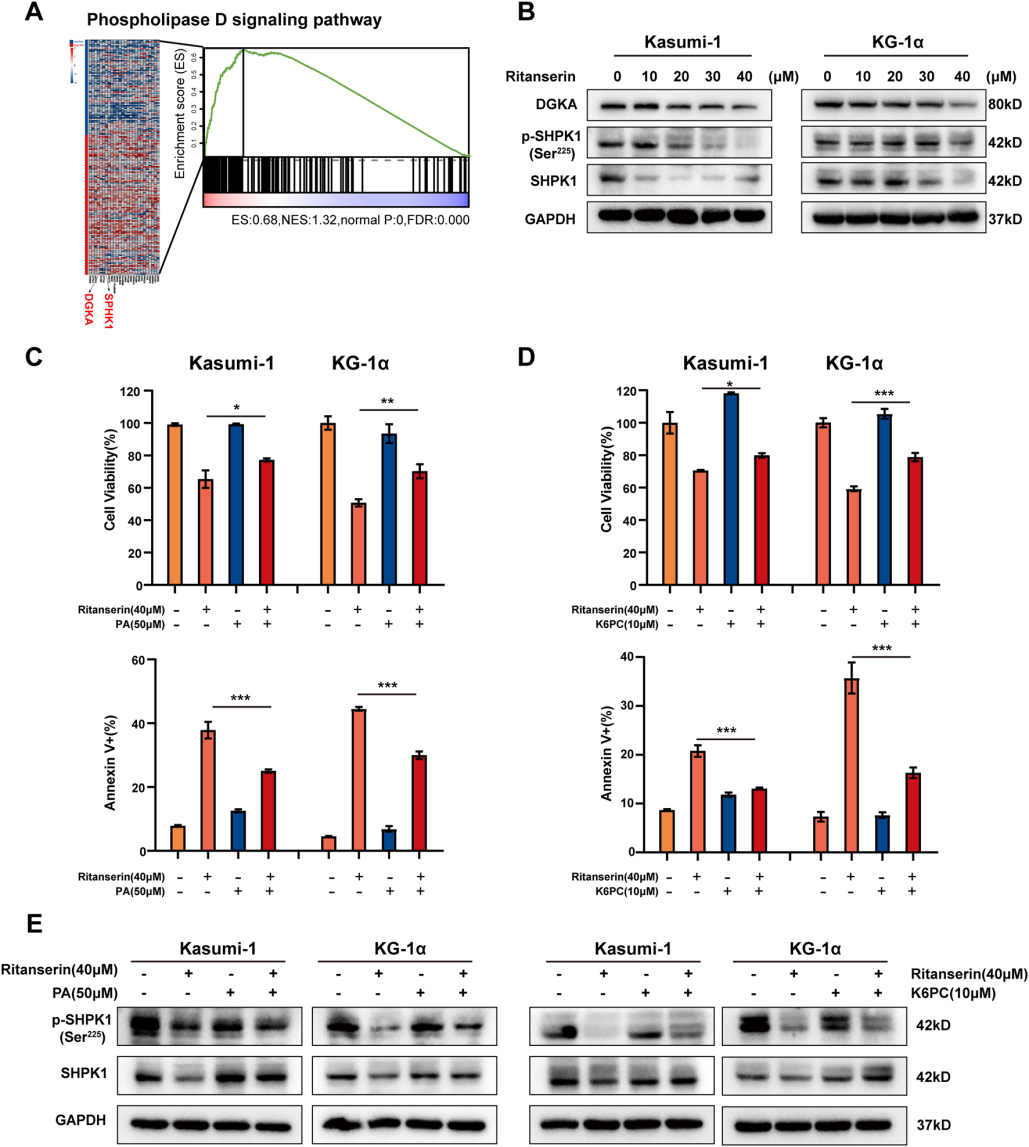
Ritanserin suppresses the phospholipase D (PLD) signaling pathway in AML cells. **A** The PLD signaling pathway is enriched in the high-risk group, of which DGKα and SphK1 are jointly involved in signal transduction of the PLD signaling pathway. **B** The protein expression levels of DGKα, SphK1 and p-SphK1(Ser225) after ritanserin treatment for 48 h. The histograms of cell viability and apoptosis cell ratio after ritanserin combined with exogenous **C** phosphatidic acid or **D** SphK1 agonist (K6PC) for 48 h. Data are presented as the mean ± S.D. of triplicate experiments. (*p < 0.05; **p < 0.01; ***p < 0.001.) **E** Western blotting indicated the expression of SphK1 and p-SphK1(Ser225) after ritanserin combined with exogenous phosphatidic acid or K6PC for 48 h

The main form of DAG metabolism is to convert DAG into phosphatidic acid (PA) through DGKα, and both DAG and PA are critical lipid second messengers in the plasma membrane [20]. Several human disorders, including cancer, have been linked to deregulated PLD-PA lipid signaling [21–23]. Pertaining to previous research and our findings, we next investigated the downstream targets of DGKα-mediated PLD signaling regulation. Indeed, DGKα and sphingosine kinase 1 (SphK1) are jointly involved in signal transduction of the PLD signaling pathway (Fig. 3A). Following ritanserin administration, we observed that the protein levels of DGKα, SphK1 and phospho-SphK1 (Ser225) were markedly diminished (Fig. 3B), which is consistent with the results of our bioinformatics analysis. These findings imply that ritanserin suppresses SphK1 expression and acts as an anti-AML agent via DGKα.

The sphingosine kinase SphK1 has been identified as a crucial signaling molecule in various growth-related cellular events, such as cell migration, proliferation, and transformation [24]. Although it has been previously established that PA can control the intracellular effector SphK1 [25], it is still unclear whether DGKα facilitates PLD-PA lipid signaling and regulates the expression of SphK1. Although ritanserin monotherapy inhibited cell proliferation and promotes apoptosis in AML cells, we discovered that exogenous PA added could rescue the suppression of AML cell growth (Fig. 3C), as well as the protein levels of SphK1 and phospho-SphK1 (Ser225) were elevated (Fig. 3E). Besides, we also introduced SphK1 agonists K6PC [26], and further confirmed that replenishment of SphK1 may increase cell viability and inhibit apoptosis (Fig. 3D). Compared with the ritanserin therapy, the expression of SphK1, phospho-SphK1 (Ser225) were also increased after K6PC treatment together (Fig. 3E). These results supported the hypothesis that ritanserin inhibits DGKα and then regulates SphK1 expression via PLD-PA lipid signaling, therefore preventing the growth of AML cells.

### Ritanserin negatively regulates the Jak-Stat and MAPK signaling pathways

**Fig. 4:**
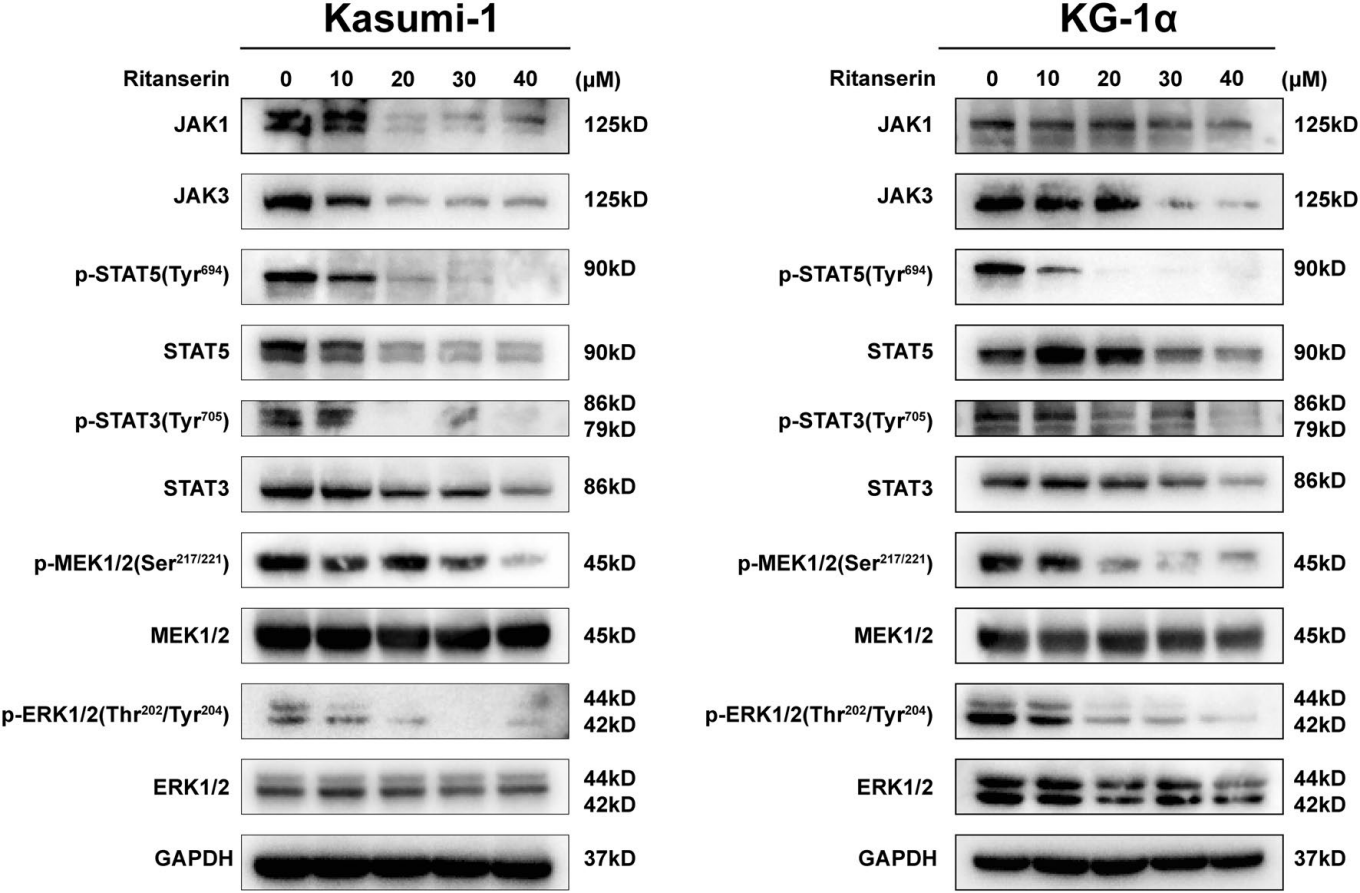
Ritanserin negatively regulates the Jak-Stat/MAPK signaling pathway. After being exposed to ritanserin for 48 h, the different proteins involving Jak-Stat/MAPK signaling pathway were detected by western blotting in Kasumi-1 and KG-1α cells

Thus far, the results have demonstrated that DGKα is essential for negative regulation of the Jak-Stat and MAPK signaling pathways, which leads to the carcinogenesis of AML (Fig. S2G, H). We further performed western blotting to discover the critical proteins for two pathways to assess the results of the transcriptome profile in TCGA. As shown in Fig. 4, the expression of total protein and the phosphorylation levels of several genes, including JAK1, JAK3, STAT3, STAT5, MEK1/2, and ERK1/2, were considerably downregulated in ritanserin-treated Kasumi-1 and KG-1α cells.

**Fig. 5:**
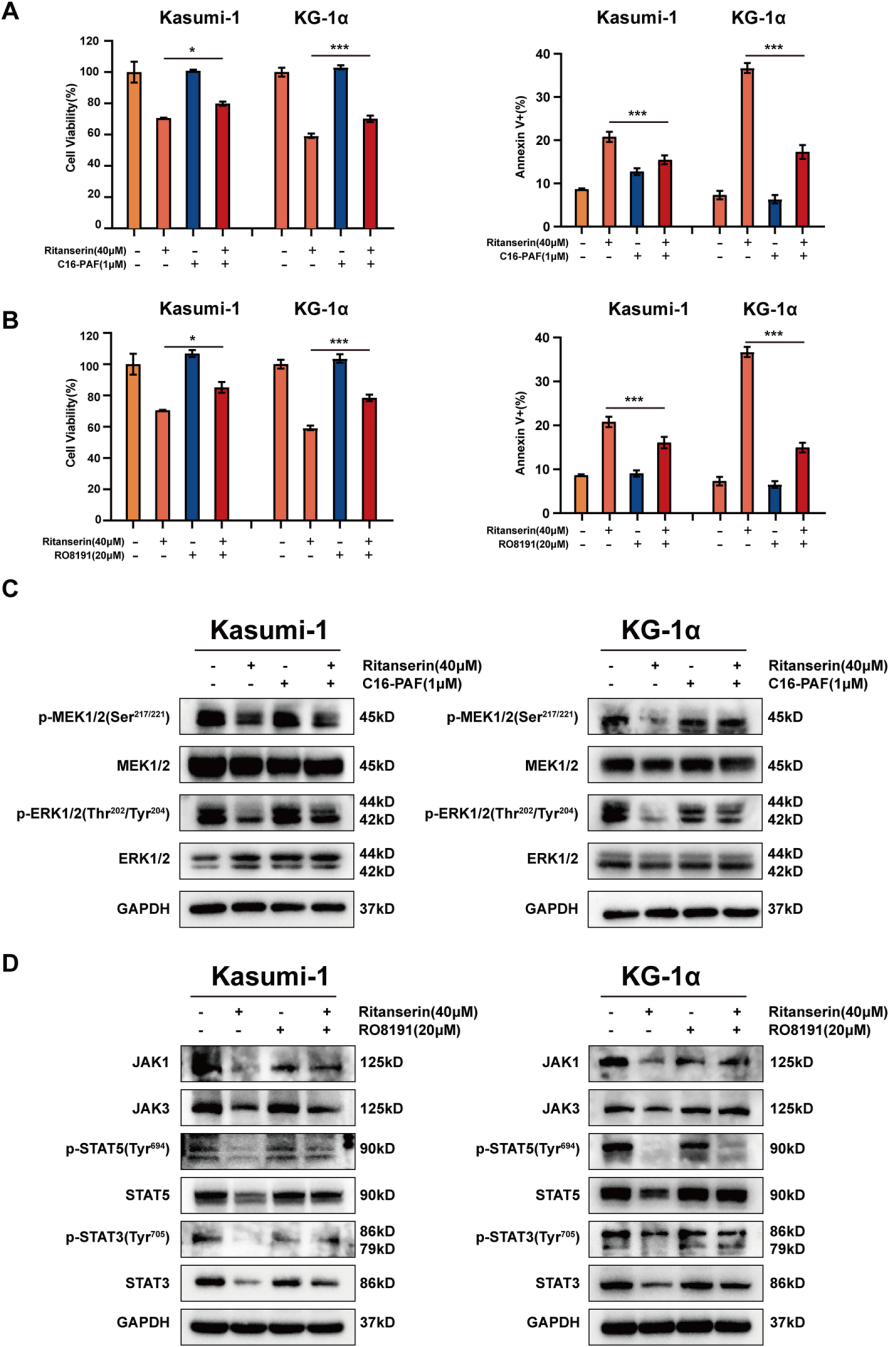
Agonists of Jak-Stat/MAPK signaling rescued the ritanserin-mediated anticancer effect and activates underlying pathways. The histograms of cell viability and apoptosis cell ratio after ritanserin combined with **A** the MAPK, MEK/ERK agonist C16-PAF or **B** the JAK/STAT agonists RO8191 for 48 h. Data are presented as the mean ± S.D. of triplicate experiments. (*p < 0.05; ***p < 0.001.) **C** The protein expression levels of p-MEK1/2(Ser217/221), MEK1/2, p-ERK1/2(Thr202/Tyr204) and ERK1/2 were assessed after treatment with ritanserin, C16-PAF or both for 48 h. **D** The protein expression levels of JAK1, JAK3, p-STAT5(Typ694), STAT5, p-STAT3(Tyr705) and STAT3 were assessed after treatment with ritanserin, RO8191 or both for 48 h

To better confirm the potential signaling pathway after ritanserin treatment, we performed rescue experiments through the potent MAPK, MEK/ERK agonists C16-PAF [27], and the JAK/STAT agonists RO8191 [28]. Compared with the ritanserin monotherapy, C16-PAF or RO8191 combined with ritanserin were revealed to increase cell proliferation and inhibit apoptosis in AML cells, accompanied by statistical differences (Fig. 5A, B). Notably, phosphorylation levels of ERK1/2 (Thr202/Tyr204) were increased after C16-PAF was added with ritanserin (Fig. 5C). The protein expression of JAK1, STAT3, STAT5, phospho-STAT3(Tyr705) and phospho-STAT5(Tyr694) were observed to elevated by co-treatment with RO8191(Fig. 5D). Thus, we deduced that the anti-leukemia efficacy of ritanserin might be influenced by DGKα-induced Jak-Stat/MAPK pathways. Together, we concluded that the anti-leukemia efficacy of ritanserin is due to its inhibition of DGKα, mainly through negative regulation of the Jak-Stat and MAPK signaling pathways.

### Ritanserin exerts an anti-AML effect in vivo

**Fig. 6:**
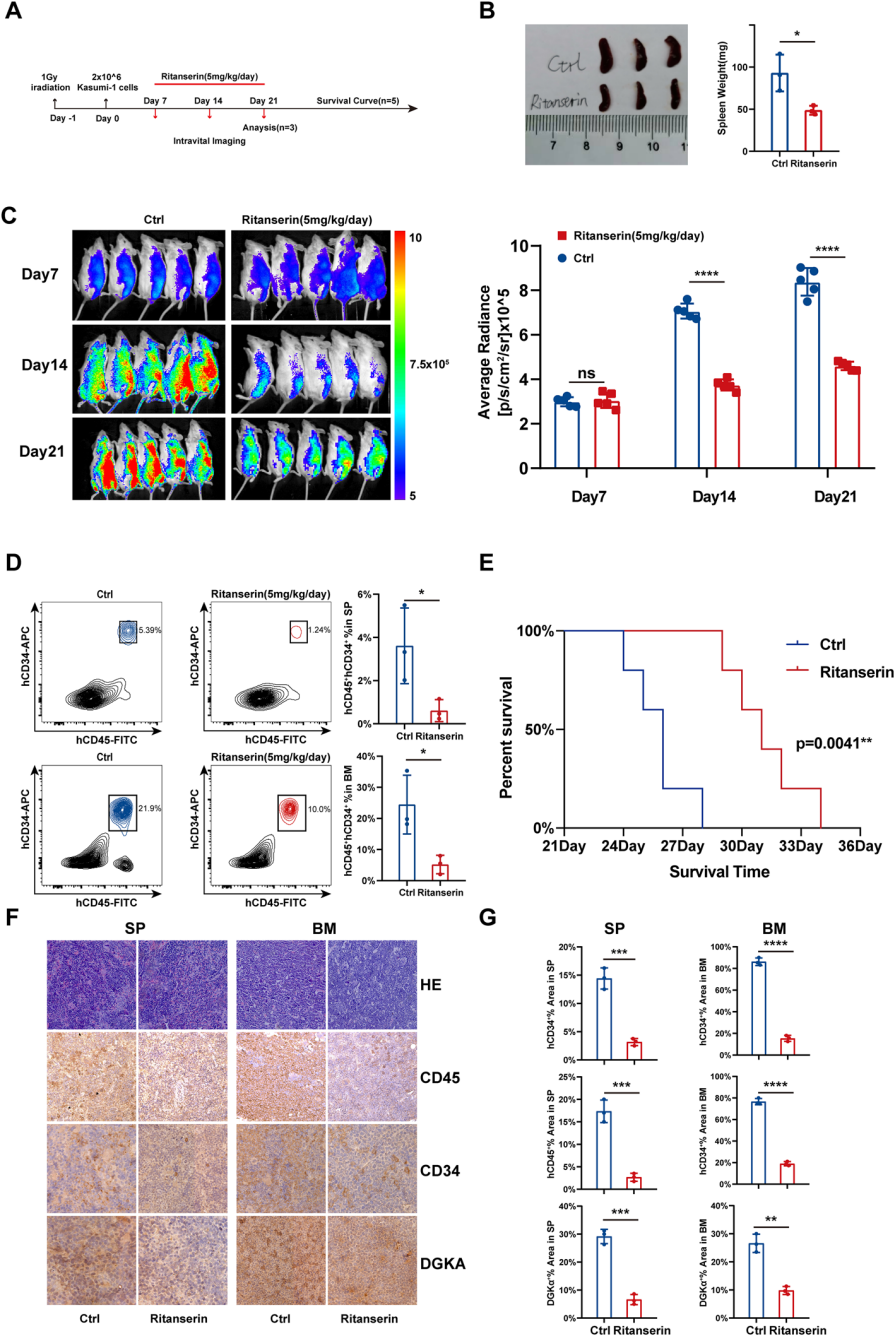
Ritanserin exerts an anti-leukemia effect in vivo. **A** Experimental protocol for the xenograft model of Kasumi-1 AML cells. **B** The spleen weight and **D** ratio of hCD45^+^ to hCD34^+^ in the spleen and bone marrow after two weeks of treatment with ritanserin (5 mg/kg/day). **C** Intravital imaging of mice treated with vehicle or ritanserin (5 mg/kg/day). The statistical graphs of Average Radiance were exhibited on the right. **E** The survival curve of leukemia xenograft mice. **F**, **G** Spleen and bone marrow were collected and stained with H&E. Immunohistochemical staining was used to detect the expression of CD45, CD34, and DGKα. Three replicates are presented as the mean ± S.D. (*p < 0.05; **p < 0.01; ***p < 0.001; ****p < 0.0001)

To evaluate whether ritanserin functioned in carcinogenesis in vivo, Kasumi-1 cells were injected intravenously into NSG mice. We randomly assigned these mice to the vehicle and ritanserin groups (5 mg/kg/day) and subjected them to 14 consecutive days of intraperitoneal treatment (Fig. 6A). On the fourteenth day following treatment, three mice were sacrificed for examination. The ritanserin-treated group exhibited considerably ameliorated AML-associated splenomegaly in comparison to the vehicle group without experiencing lethal effects (Fig. 6B, Fig. S3A). The expression of CD34 and CD45 was assessed using flow cytometry to further pinpoint leukemia infiltration in vivo. The leukemia burden in the mouse spleen (SP) and bone marrow (BM) was potently attenuated by ritanserin (Fig. 6D, Fig. S3B). Most importantly, ritanserin substantially decreased leukemia carcinogenesis in vivo and along with significant statistical differences (Fig. 6C). Ritanserin was also determined to effectively prolonged survival (Fig. 6E). Additionally, immunohistochemical staining also revealed that ritanserin significantly reduced the expression of CD34, CD45, and DGKα (Fig. 6F, G). In general, ritanserin hindered AML carcinogenesis in vivo.

### Ritanserin promotes primary cell apoptosis in AML

**Fig. 7:**
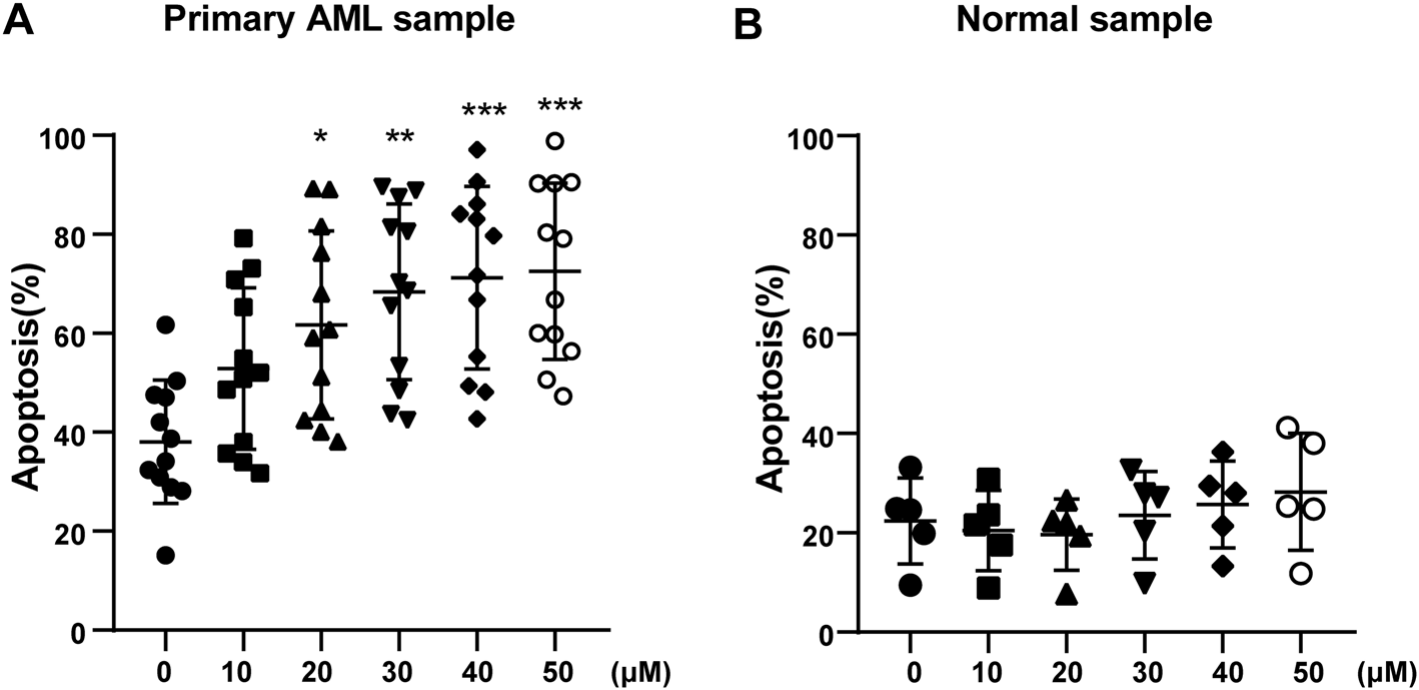
Ritanserin induces cell apoptosis in primary AML samples. After treatment with ritanserin for 24 h, Annexin V/PI double staining was employed to detect the apoptosis ratio in **A** primary AML cells and **B** normal PBMCs

Finally, we collected bone marrow mononuclear cells (BMMCs) from 12 primary AML samples to investigate the clinical application of ritanserin. The clinical characteristics of all samples are summarized in Table 2. After treating primary samples for 24 h, ritanserin induced primary cell apoptosis and exhibited substantial toxicity in AML (Fig. 7A). These results are in agreement with the in vitro data from experiments in cell lines. In contrast, ritanserin showed minimal toxicity to samples of normal hematopoietic stem cells (Fig. 7B), suggesting that ritanserin could be prescribed for targeted therapy of AML. Overall, these results confirmed that ritanserin has preclinical anti-leukemia capability.

**Table 2:** Clinical characteristics of AML patients

Patient No	Gender	Age	FAB	WBC (× 10^9^ /L)	Molecular mutations
1	M	44	M5	62.39	WT1,FLT-ITD,NPM1,DNMT3A
2	M	64	M2	28.67	WT1/ABL, ASXL1,DNMT3A, RUNX1, SRSF2,TP53
3	M	50	M4E0	65.37	CBFbeta-MYH11, c-kit,RAD21
4	F	75	M5	29.03	WT1, DNMT3A,IDH1,NPM1
5	F	57	M3	11.46	BCOR, TET2,PML-RARa
6	M	69	M5	23.42	WT1, EVI1,FLT3-TKD,RUNX1,SF3B1
7	M	65	M4E0	53.27	–
8	M	18	Unclassified	38.09	BCR-ABL
9	M	42	M5	53.27	MLL-AF6, EV11
10	M	28	Unclassified	38.09	–
11	F	38	M5	0.34	DNMT3A, IDH1,NPM1
12	M	18	M2	25.79	–

## Discussion

The refractory and relapsed forms of AML usually result in death; nevertheless, there are few effective treatment options for R/R AML [29]. Allogeneic hematopoietic stem cell transplantation (HSCT) is only used for the treatment of AML patients. However, due to being unfit or other factors, only a small number of patients are eligible to receive allo-HSCT [1]. Beyond this guiding principle, for most patients R/R AML patients, both older and younger, a consistent course of therapy is lacking. New insights into several novel small molecule inhibitors provide the opportunity to revisit the treatment approach for R/R AML. In this study, for the first time, we evaluated the cytotoxicity and underlying mechanisms of the DGKα inhibitor ritanserin in AML preclinical models.

The discovery of ritanserin as a DGKα inhibitor highlights the value of repurposing medications, as previous clinical trial data established its safety and tolerability in human subjects [30]. Studies have shown that ritanserin inhibits C-RAF to cause apoptosis in lung cancer cells and prevents glioblastoma multiforme (GBM) and pancreatic cancer spread by modifying DGKα, which promotes the mesenchymal phenotype [10, 12]. Herein, we provide evidence that ritanserin inhibited AML tumor growth. Ritanserin exhibited dose-dependent cytotoxicity at therapeutically feasible doses, and different cells had varied responses to ritanserin. Ritanserin generated a higher level of apoptosis in KG-1α cells than in Kasumi-1 cells. This is supported by the stimulation of caspase-3 and PARP, which may be due to unknown mutations in cell origin or gene development. We also determined the clinical value of ritanserin: it effectively suppresses primary leukemia cells and considerably prolongs survival in mouse xenograft models. Overall, ritanserin provides a potent anti-leukemia effect.

The expression of DGKα is increased in several cancer cells with poor prognosis, such as hepatocellular carcinoma, melanoma and glioblastoma [9, 31, 32]. It is widely known that DGKα phosphorylates DAG to produce PA and that PA generated by DGKα is essential for the growth and anti-apoptotic properties of cancer cells [20, 33]. Meanwhile, studies have shown that DGKα is highly expressed in the nucleus of human erythroleukemia K562 and promotes cell proliferation and cell cycle progression [19]. After knocking down DGKα, it was found that the proliferation of K562 cells was inhibited [19], and similar effects were observed in lymphocytes treated with DGKα inhibitors [34]. We discovered that DGKα expression is elevated in AML using the TCGA and GTEx datasets and verified its function in our cohort. Our results also showed that a poorer prognosis is predicted by high DGKα expression. All of these findings are in line with other studies and clearly imply that DGKα may be a therapeutic target of broad interest and promise in the hematological system, particularly in AML. Additionally, we discovered that CD34 was highly expressed in high-risk groups and positively correlated with DGKα expression. The recurrence of AML shows that targeted leukemia stem cells (LSCs) therapy is still flawed. This rare drug-resistant cell is responsible for maintaining leukemia and is usually enriched in CD34^+^CD38^−^cells [35, 36]. Consequently, high expression of DGKα may indicate a high CD34 level, consistent with a poor prognosis for AML.

Despite the progress in linking the function of DGKα with the development of cancer and other diseases, it is still challenging to study their biology. After determining the crucial function of DGKα in AML, we carried out a pathway enrichment study. Notably, DGKα mostly activates the PLD pathway and associated pathways in AML samples. PA functions as a second signal to control SphK1 expression [25], and DGKα and SphK1 overexpression in AML are positively correlated. We discovered that ritanserin inhibits DGKα and further reduces the phosphorylation of SphK1. Replenishment of exogenous PA and SphK1 agonists K6PC rescued cell proliferation and apoptosis, which is in line with previous research showing that decreased SphK1 expression facilitates Mcl-1 degradation and increases apoptosis [37]. In addition, the DEGs in the DGKα high-risk group were enriched in the Jak-Stat/MAPK signaling pathway, whose activation induces partial AML production [38] and promotes tumor growth [39], and some AML patients are resistant to conventional treatment due to MAPK pathway activation [40]. This study confirmed that these pathways were inactivated after treatment with the DGKα inhibitor ritanserin, and rescued by the MAPK, MEK/ERK agonists C16-PAF, and the JAK/STAT agonists RO8191. Taken together, our results, although preliminary, indicate that ritanserin can regulate PLD-related pathways and the Jak-Stat/MAPK signaling pathway to prevent the progression of AML.

## Conclusion

In conclusion, we found that selective inhibition of DGKα by ritanserin inhibits AML cell lines and primary patient cells both ex vivo and in in vivo mouse xenografts as a single agent in the clinically achievable range. Moreover, we determined that DGKα may be a promising therapeutic target in AML, and ritanserin not only negatively regulates SphK1 expression through PLD signaling but also inhibits the Jak-Stat and MAPK signaling pathways via DGKα, providing effective preclinical evidence for ritanserin in the treatment of AML.

## Supplementary Information

Supplementary file1

Supplementary file2

Supplementary file3

## Data Availability

The data presented in this study are available on request from the corresponding author.

## Abbreviations

AML: Acute myeloid leukemia
R/R: Refractory or relapsed
OS: Overall survival
CR: Complete remission
DAG: Diacylglycerol
DGKα: Diacylglycerol kinase alpha
PLD: Phospholipase D
GO: Gene ontology
KEGG: Kyoto encyclopedia of genes and genomes
GSEA: Gene set enrichment analysis
PA: Phosphatidic acid
SphK1: Sphingosine kinase 1
BMMCs: Bone marrow mononuclear cells
HSCT: Hematopoietic stem cell transplantation
LSCs: Leukemia stem cells
MAPK: Mitogen-activated protein kinase
DMSO: Dimethyl sulfoxide
SD: Standard deviation
SP: Spleen
BM: Bone marrow
GBM: Glioblastoma multiforme
