# Supplementary figures and images for: Ritanserin suppresses acute myeloid leukemia by inhibiting DGKα to downregulate phospholipase D and the Jak-Stat/MAPK pathway
Source: Discov Oncol. 2023 Jul 1;14:118. doi: 10.1007/s12672-023-00737-9 (PMC10314883; doi:10.1007/s12672-023-00737-9)

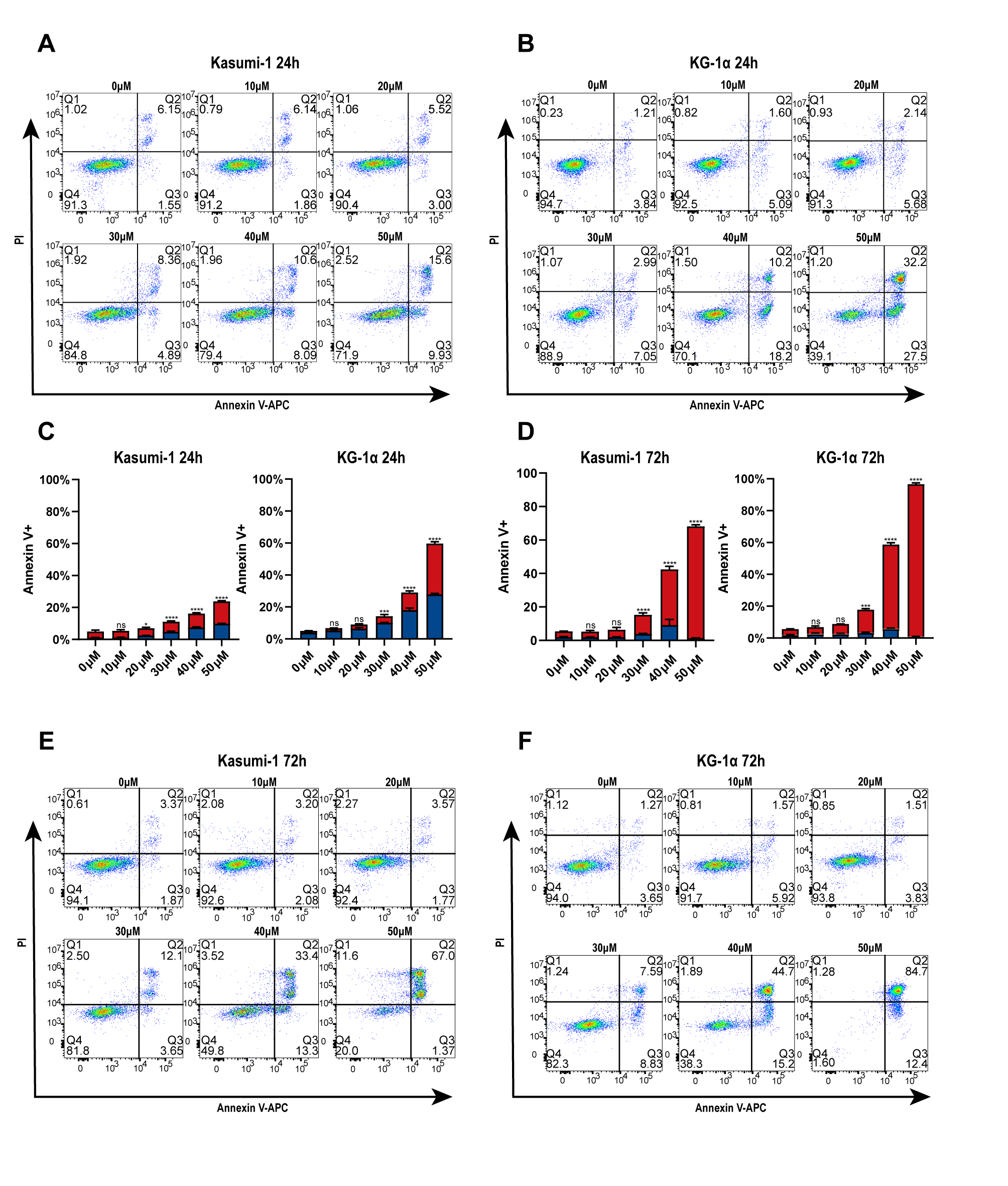

Supplement: Supplementary file 1 — Supplementary file1 [file 12672_2023_737_MOESM1_ESM.tif]

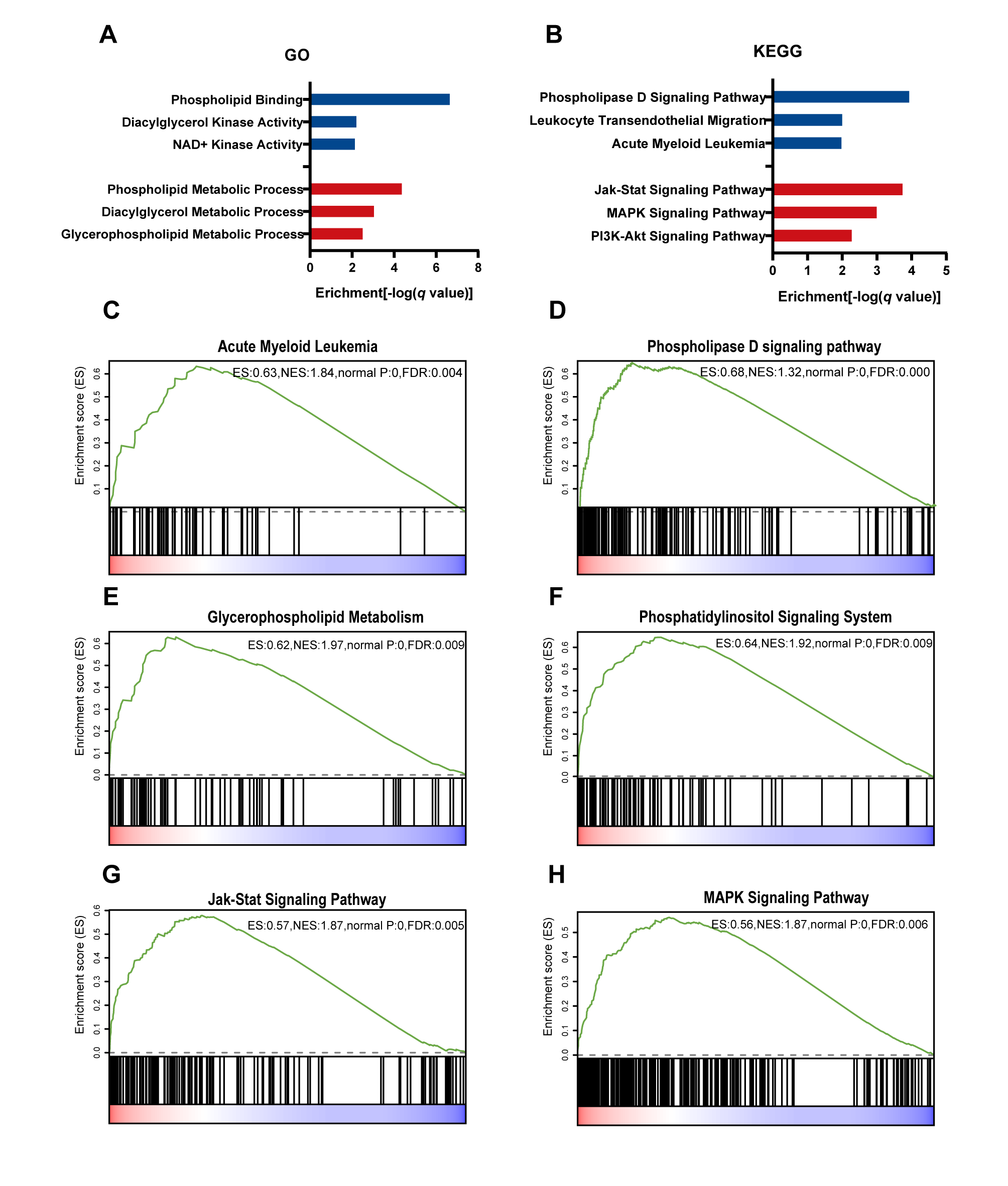

Supplement: Supplementary file 2 — Supplementary file2 [file 12672_2023_737_MOESM2_ESM.tif]

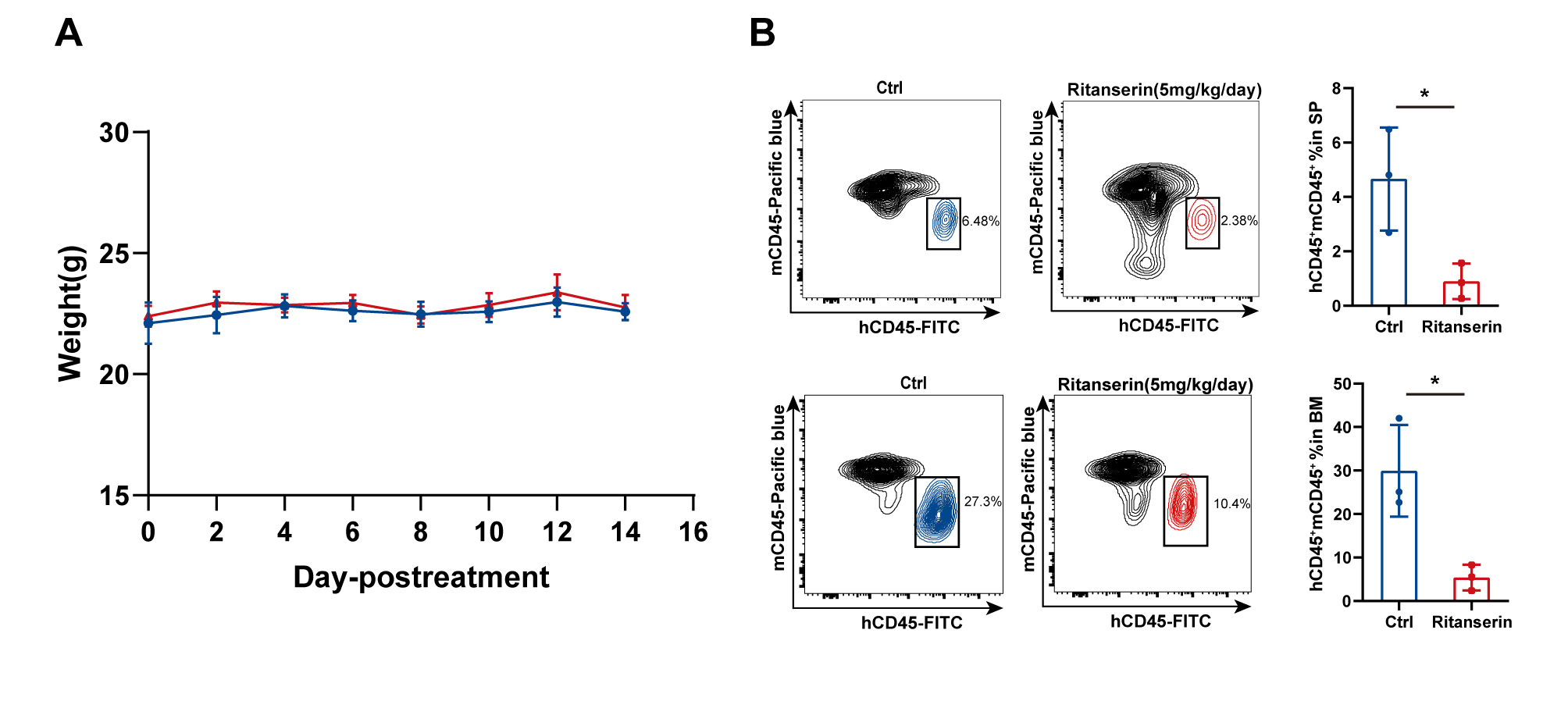

Supplement: Supplementary file 3 — Supplementary file3 [file 12672_2023_737_MOESM3_ESM.tif]
